# Effectiveness and mechanism of action of rTMS combined with quadriceps strength training in individuals with knee osteoarthritis: study protocol for a randomized controlled trial

**DOI:** 10.1186/s12891-023-07146-7

**Published:** 2024-01-05

**Authors:** Ming-Hui Lai, Hai-Chen Xu, Yu-Wu Ding, Kun Yang, Xue-Ping Xu, Li-Ming Jiang

**Affiliations:** Department of Rehabilitation, Seventh People’s Hospital of Shanghai University of Traditional Chinese Medicine, Datong Rd. 358, Shanghai, 200137 China

**Keywords:** Knee osteoarthritis, High-frequency rTMS, Quadriceps, Mechanism of action

## Abstract

**Background:**

Quadriceps training is necessary in function and activity of daily living for patients with knee osteoarthritis (KOA). However, it did not reduce the rate of surgical treatment for end-stage KOA in the long term. This may be related to brain structure changes and maladaptive plasticity in KOA patients. Transcranial Magnetic Stimulation (TMS) could enhance the functional connectivity of brain regions and improves maladaptive plasticity. However, the synergistic effect of the combination of the two for treat KOA is still unclear. Therefore, the purpose of this study is to investigate whether the High-Frequency rTMS combined with quadriceps strength training can improve the pain and function in KOA more effectively than quadriceps training alone and explore the mechanism of action.

**Methods:**

This study is an assessor-blind, sham-controlled, randomized controlled trial involving 12 weeks of intervention and 6 months follow-up. 148 participants with KOA will receive usual care management and be randomized into four subgroups equally, including quadriceps strength training, high-frequency rTMS training, sham rTMS and quadriceps strength training, high-frequency rTMS and quadriceps strength training. The rehabilitation interventions will be carried out 5 days per week for a total of 12 weeks. All outcomes will be measured at baseline, 4 weeks, 8 weeks, and 12 weeks during the intervention and 1 month, 3 months and 6 months during the follow-up period. The effectiveness outcomes will be included visual analog scale, isokinetic knee muscle strength, Knee Injury and Osteoarthritis Outcome score and 36-Item Short-Form Health Survey score; The act mechanism outcomes will be included motor evoked potential, grey matter density, white matter, subcortical nuclei volumes, cortical thickness and functional connectivity by MRI. Two-way of variance with repeated measures will be used to test the group and time effect for outcome measures.

**Discussion:**

The study will be the first protocol to examine whether there are synergistic effects following high-frequency rTMS combined with quadriceps strength training for treat KOA and clarify the mechanism of action. High-frequency rTMS can be added into the training program for KOA patients if it is proven effective.

**Trial registration:**

Chinese Clinical Trial Registry ChiCTR2300067617. Registered on Jan.13,2023.

## Background

Knee osteoarthritis (KOA) is a chronic degenerative joint disease characterized by degenerative cartilage lesions and secondary bone hyperplasia of the knee joint [1]. KOA accounts for more than 80% of total osteoarthritis and is the leading cause of disability, decrease quality of life in the adult and elderly population [2]. The management of KOA may be requiring long-term treatment, leading to significant financial strain on individuals, families, and society as a whole. Consequently, it is imperative to investigate and find effective therapeutic approaches for individuals with KOA, benefiting both the affected individuals and society at large.

To date, there is no proven treatment method that can reverse the course of KOA [3]. Current therapeutic strategies for KOA are focus on improving muscle strength or relieving pain, including medication, physical exercise, intra-articular injection, etc. Clinical guidelines recommend physical exercise as one of the most critical non-pharmacological treatments of KOA [4], which has been proven to improve knee function effectively [5]. Quadriceps strengthening is an critical component of the exercise program because it has been found that patients with KOA always have significant quadriceps weakness [6]. Previous studies indicate quadriceps strengthening is benefit for the relief of knee joint pain and stiffness and improving quality of life [7–9]. Although exercise is effective in KOA, a recent meta-analysis indicated its treatment benefits are moderate for pain and function, performance at or nearest to 8 weeks. Besides, the exercise effects appeared to peak around 2 months and then gradually decreased and became no better than usual care after 9 months [10]. It means the effects of exercise will plateau and pain always persist. Leandro et al. [11] also have proved that exercise treatment effectiveness and adherence seem to decrease over time.

In recent years, more and more evidence has proved that low activation of M1 [12] and secondary to gamma loop [13] and motor-sensory circuit disorders [14] may be potential reasons for weakness of quadricep muscles in KOA. It can be seen that the enhancement of muscle strength for KOA is not only about the improvement and optimization of muscle strength training program, but also about the activation of brain areas. Therefore, the M1 may be a potential neurotherapeutic target for therapy in KOA [15]. Furthermore, another study showed that chronic pain in KOA can also further increase motor cortex inhibition. The chronic pain in KOA patients is not necessarily related to damage to the peripheral muscular system, but rather to neuroplastic changes in pain-related circuits that result in maladaptive neuroplasticity and lead to a perpetuation of pain [16]. The neuroplasticity changes in response to constant peripheral pain and less intracortical inhibition, which leads to enhance the levels of pain and dysfunction [17, 18].

Non-invasive brain stimulation is an increasingly growing domain of rehabilitation therapeutics for clinical neurological disorders. A variety of techniques for transcranial brain stimulation has been proposed, such as, Transcranial Direct Current Stimulation (tDCS), Transcranial Magnetic Stimulation (TMS) [19]. A general opinion is that modulation of neuronal excitability by tDCS is a relatively simple function (e.g., cortical excitability is reduced by cathodal stimulation and increased by anodal stimulation) and tDCS effects are difficult to predict, rarely used to treat diseases with various neural circuits, such as, pain related to central sensitization [20]. For rTMS, the magnetic field pulse delivered by a stimulating coil applied on the scalp is able to pass through skull bone without being attenuated and to generate an electric field when entering the brain. The intensity of the induced current is sufficient to produce action potentials and to activate brain networks safely and painlessly [21]. rTMS has been proposed to treat various pain syndromes related to central sensitization phenomenon, a Level A of definite analgesic effect was stated for the use of high frequency rTMS of M1 applied contralaterally to the pain side in patients with neuropathic pain. Previous study demonstrated that the chronic pain associated with KOA may develop in connection with a maladaptive process of pain sensitization in the central nervous system [22]. Hence, there is a basis for speculation that rTMS could be beneficial in alleviating pain associated with KOA.

Although the positive rehabilitation effect of quadriceps strengthening and high-frequency rTMS for KOA have been demonstrated, respectively, the synergistic effect of the combination of the two for treat KOA is still unclear. Therefore, the randomized controlled trial is designed to observe whether high-frequency rTMS combined with quadriceps strength training can better improve the knee function and reduce pain in KOA patients than quadriceps strength training alone and explore the mechanism of this combined rehabilitation. The study will provide a new perspective on the treatment of KOA.

## Methods

### Study design

**Fig. 1:**
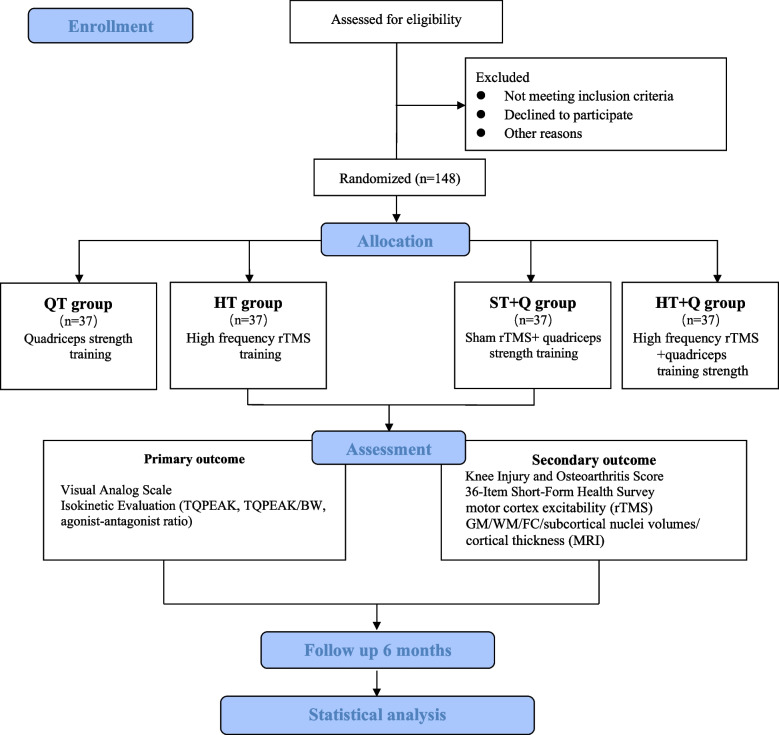
A brief flowchart of the entire study. TQPEAK, peak torque; TQPEAK/BW, TQPEAK adjusted for body weight; rTMS, repetitive transcranial magnetic stimulation; GM, grey matter density; WM, white matter; FC, functional connectivity; MRI, Magnetic resonance imaging

This study will be a randomized controlled trial with a parallel-group design. A total of 148 eligible participants will be assigned to the quadriceps strength training (QT group); high frequency rTMS (HT group); sham rTMS and quadriceps strength training (ST + Q group); high frequency rTMS and quadriceps strength training (HT + Q group) at 1:1:1:1 ratio using stratified randomization with gender, age and Kellgren-Lawrence (KL) grade as factors. A brief flowchart of the entire study is shown in Fig. 1 and the schedule of events is provided in Table 1. The study protocol was approved by the Ethics Committee of Shanghai Seventh People’s Hospital (2022-7th-HIRB-065) and registered in the Chinese Clinical Trial Registry (ChiCTR2300067617).

**Table 1:**
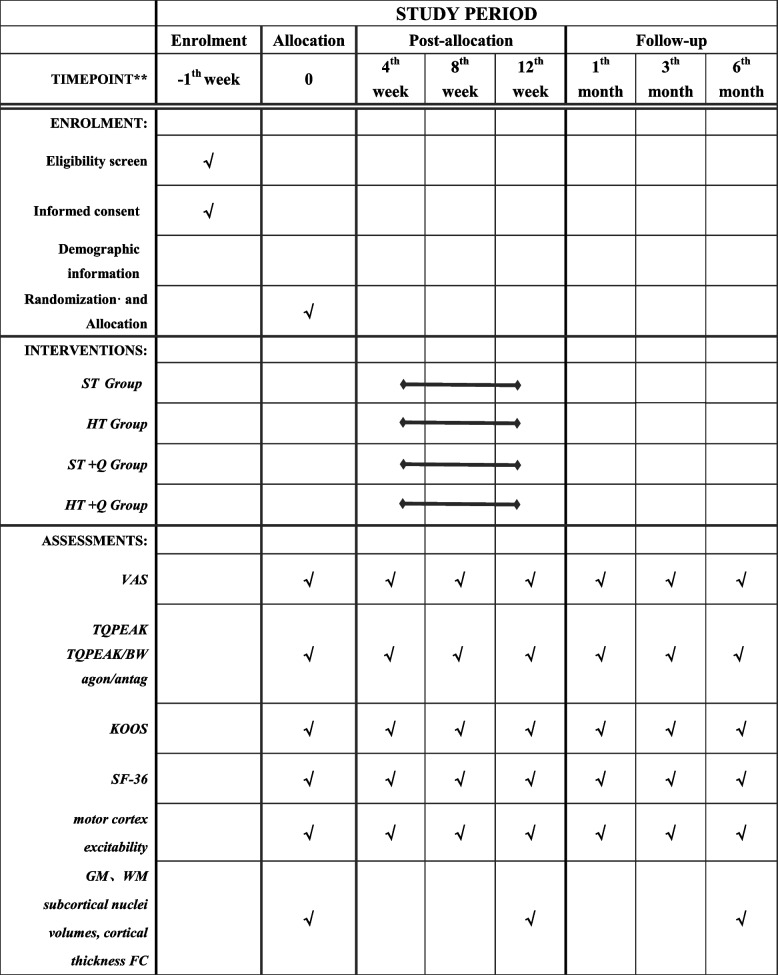
Schedule of enrolment, intervention and assessments

"√" means things will be done

*VAS* Visual Analog Scale, *TQPEAK* peak torque, *TQPEAK/BW* TQPEAK adjusted for body weight; agon/antag, agonist–antagonist ratio, *KOOS* Knee Injury and Osteoarthritis Outcome Score, *SF-36* 36-Item Short Form Survey, *GM* grey matter density, *WM* white matter, *FC* subcortical nuclei volumes, cortical thickness; functional connectivity

### Sample size calculation

The sample size was determined based on the priori power analysis through G*Power software version 3.1.9.2. OMERACT-OARSI hip and knee osteoarthritis core outcome set [23] showed that the pain is the most core outcome and the percentage frequency is 94.8% from 1997 to 2017. Furthermore, Guinot et al. [24] reported that the effect size of rTMS for improve pain score in patients with fibromyalgia was 0.24. Therefore, the effect size was set as 0.24 in the present protocol. In the calculations, ANOVA repeated measures between factor was chosen, effect size 0.24, alpha 0.05, power 0.80, number of groups 4, number of measures 6, and the sample size was calculated as 116. Taking into account a conservative anticipation of a 20% drop-out rate, the final sample size will therefore be 37 in each group, with a total of 148 subjects.

### Participants

#### Inclusion criteria

1. Meet the diagnostic criteria for KOA set by the ACR in 2019 [25]
2. Age between 40 and 60 years old
3. Kellgren-Lawrence (KL) grade II to III based on radiographic classification [26]
4. Have a minimum score of 25 on the Western Ontario and McMaster Universities Osteoarthritis Index (WOMAC) total score
5. Sign an informed consent form

#### Exclusion criteria

1. Knee or hip arthroplasty
2. Any uncontrolled non-musculoskeletal conditions that would make testing difficult and uncomfortable, such as unstable heart conditions
3. Neurological condition that affects lower-limb strength or walk (e.g., stroke)
4. Have a contraindication for TMS use such as: the existence of metal in the skull or implanted devices, epilepsy history, pregnancy
5. Use of drugs that might affect cortical electrical activity (anticonvulsants, antidepressants or antipsychotics)
6. Have a contraindication for MRI use, such as: pacemaker, metal implant etc.

#### Drop-out criteria

1. Inability to return for treatment for any reason
2. Adverse events occur due to treatment
3. Participants who receive other interventions to treat KOA or others that may interfere with the results of this study (e.g., using intra-articular or oral corticosteroids; post-surgical knee, knee or hip joint replacement surgery, tibial osteotomy; participating in regular physical exercise, such as swimming, cycling, etc.)

### Recruitment, enrollment and randomization procedures

All patients with KOA will be recruited from the orthopedics or joint surgery department of Shanghai Seventh People’s Hospital in China and communities around the hospital. Recruitment methods will include posters, online advertisements, and leaflets. We will communicate with prospective participants about the study details. After the patients voluntarily sign the informed consent, they will be invited to participate in the study. Recruitment will start from 1 October 2023, until when 148 participants are enrolled.

A total of 148 eligible participants will be assigned to four groups at a 1:1:1:1 ratio using stratified randomization with gender, age and KL grade as factors in this research. The stratified randomization will be achieved as follows: Firstly, participants will be invaded into men and women two groups. Secondly, the two groups will be grouped by age (< 50 and ≥ 50). Lastly, the four groups will be grouped by KL grade (II and III), a total of 8 subgroups. In these eight subgroups, the subjects will be randomly regrouped the QT group, HT group, ST + Q group and HT + Q group. Finally, all four groups will be regrouped into four new groups to minimize the bias of the final results. The randomization procedure is performed by an independent research assistant who is not comes into contact with the participant at any point prior to randomization and is not involved in the data collection using Microsoft Excel to generate the random number (https://www.microsoft.com).

### Allocation concealment and blinding

Allocation concealment will be performed by using sealed, opaque envelopes which will hide a serial number. Additionally, an independent study researcher will open the envelopes in sequence after participants complete all baseline assessments to avoid selective bias. Due to the visibility of the quadriceps strength training intervention, physiotherapists and patients cannot be blind to intervention allocation. Therefore, the blinding will only be applied to the assessors and statisticians who responsible for data collection and final statistical analyses in this study to avoid implementation bias and measurement bias. Importantly, they will not participate in the participant recruitment process.

### Interventions

All participants will receive usual care management, including health education and physical therapy when necessary. Six qualified physiotherapists who have attained the physical therapist’s certification with over 5 years of experience will be trained beforehand to instruct and guide patients in carrying out these exercises. The interventions in four groups will be carried out 5 days per week for a total of 12 weeks.

### QT Group

In the QT group, the participants will receive 20 min quadriceps strength training intervention by using Biodex Multi-Joint System 3 dynamometer (Biodex Medicalt, Shirley, NY, USA). The 12- week intervention duration period has been chosen based on evidence that at least 12 sessions of supervised exercise are required for exercise to be effective in KOA, with a number of studies demonstrating symptom improvement in KOA after 8–12 weeks of exercise [27]. Thus, 12 weeks should be sufficient to show improvement in this population. We will modify the program according to the user’s manual to reduce the risk to participants and check the device for calibration errors. All participants will be informed how the test will be and its purposes. They will conduct two familiarization sessions before the formal intervention to ensure that they feel comfortable during the exercises and perform the technique correctly. After familiarization sessions, participants will be instructed to sit in an isokinetic dynamometer and the V elcro® fixation straps will be tied around the chest, hip and distal thigh of the training limb to prevent compensatory motion from occurring. The dynamometer axis will be aligned with the canter of the lateral femoral condyle. The lever arm will be adjusted according to the training leg length and the resistance will be applied anterior to the ankle joint. The training knee will be kept at a 90° flexed position and they will be instructed to extend knee at angular velocities of 60°/sec, 90°/sec and 120°/sec with 15 repetitions in 3 sets. Rest periods of 30 s between each test extension and 2 min between each velocity will be given. Training will be performed 5 days per week for 12 weeks. A regular physiotherapist will conduct all isokinetic testing with verbal stimuli. Outcome parameters will be assessed by an independent evaluator, who is experienced in handling isokinetic devices [28]. The peak torque (TQPEAK), TQPEAK adjusted for body weight (TQPEAK/BW) and agonist–antagonist (agon/antag) ratio will be the outcomes.

### HT Group

In the HT group, the participants will receive 20 min high-frequency rTMS training intervention. The rTMS will be performed with a Super-Rapid Magstim Stimulator (The Magstim Co., Whitland, UK), including a figure-8 type coil. The reason why we chose the figure-8 type coil is not only because it has a stronger focus, but it reduces the risk of seizures and other side effects than the h-coil and circular type coil [29]. All of the rTMS program will be performed as the recommendations of the International Federation of Clinical Neurophysiology [30, 31]. The coil will be placed in the M1 area on the opposite side of the pain and uniformly in the left when the patient has bilateral knee pain, as in previous rTMS studies [32, 33]. To determine the coil position, we first will place the center of the helmet to install the figure-8 type coil at a point 1 cm lateral and 1 cm posterior from Cz. Then, we will identify the location and angle of the helmet by identifying the minimum stimulator intensity needed to cause a motor response in the targeted lower limb. We will keep the front surface of the helmet facing forward to ensure that the coil orientation is the same. The resting motor threshold (rMT) will be defined as the lowest intensity that produced five responses with peak-to-peak amplitude of at least 50mv in ten consecutive trials. We will determine the coil position and estimation of MT before the high-frequency rTMS session and the parameters are as follows: 10 Hz stimulation for 4 s per session, with a 40 s interval between sessions, 30 sessions per treatment, totaling 1200 pulses at 90% rMT over M1 of the target hemisphere.

### ST + Q Group

In the ST + Q group, sham rTMS will be delivered with the coil angle rotated 90° and only one wing of the coil touching the scalp of the participant to avoid inducing actual stimulation. The parameters, including noise, time, and frequency of the sham rTMS, will be the same as HT groups [34]. Each patient will receive sham rTMS daily for five consecutive days.

### HT + Q Group

In the HT + Q group, the participants will receive active High-frequency rTMS and quadriceps strength training intervention with randomization of treatment order. The parameters, including noise, time, and frequency of the High-frequency rTMS, will be the same as HT groups, and the detail of quadriceps strength training will be the same as described above.

### Outcomes

Participants will be assessed by other physiotherapists blinded to the group allocation at different time points based on different assessments shown in Table 1. Additionally, baseline age, gender, symptoms, disease severity, duration, previous treatment and medication will be recorded using a questionnaire (in week 0). The primary end-point is the 12 weeks and all side effects during the study also will be recorded in real time.

### Effectiveness

#### Primary outcomes: VAS

The Visual Analog Scale (VAS) will be used as one of the primary outcomes to evaluate the improvement of knee pain, which consists of a 10-cm line, where 0 represents “no pain” and 10 represents “worst possible pain”. The participants will be asked to recall knee pain related to knee joint movement in the previous week and mark on the line. The reliability and validity of the VAS in application of musculoskeletal conditions is proved good [35]. In order to determine the clinical significance of the change in VAS score, the minimal clinically important difference (MCID) will be employed, and the MCID set as 1.16 based on previous study [36].

#### Secondary outcomes: Muscle strength

The maximal isokinetic muscle strength will be assessed by the Biodex Multi-Joint System 3 dynamometer (Biodex Medicalt, Shirley, NY, USA). Isokinetic exercise is a mode of speed-constant training, that can be used at low, moderate and high velocity for different evaluations and rehabilitation programs and provides reliable data. It is actually always used to quantify muscle strength, treatment and rehabilitation efficacy with mechanical or neurological instability of the knee or ligament injury [37].

#### Secondary outcomes: Knee Injury and Osteoarthritis Outcome Score (KOOS)

The Knee Injury and Osteoarthritis Outcome Score (KOOS, http://www.koos.nu, Chinese version) is a questionnaire that is self-administered and participants need approximately 10 minutes to answer all questions [38]. Different from Western Ontario and McMaster Universities Arthritis Index (WOMAC), KOOS has a more comprehensive assessment including five subscales: pain, symptom (stiff), activity of daily living, physical function, quality of life. Every question has a minimum score of 0 and a maximum of 4 points. After the score of each part is calculated separately, it is converted into a percentage score by the conversion formula. 0 points of the converted percentage score mean that the function of this part of the joint is very poor, while 100 points means that the function of this part of the joint is completely normal [39]. The KOOS shows good validity and reliability for patients with mild to moderate KOA [40]. The MCID of the KOOS score set as 10 based on previous study [36].

#### Secondary outcomes: 36-Item Short-Form Health Survey (SF-36)

The 36-Item Short-Form Health Survey (SF-36, Chinese version) is the secondary outcome measure which is a health-related questionnaire, and used to assess the quality of life (QOL) with high reliability and validity [41]. The scale is divided into physical health QOL and mental health QOL and includes 36 questions in total. The former consists of physical function, social function, physical role function and emotional role function, The latter contains mental health, energy fatigue, pain, and general health. The scale assesses QOL over the past month and the score of the SF-36 is 0–100 with higher scores indicating better QOL. [42]. The MCID of the SF-36 score set as 2.5 based on previous studies [43].

### Mechanism of action

#### Primary outcomes: motor cortex excitability

Transcranial magnetic stimulation (TMS) is a non-invasive brain stimulation tool and commonly used to measure the functional level of cortex excitability and physiology in vivo [44–46]. The motor evoked potential (MEP) is an important index marker of corticospinal excitability which means when magnetic stimulation over M1, the descending pathway produces excitability and contralateral muscle contraction of the recorded value. The cortical silent period (CSP) represents momentary suppression of MEP due to GABAergic. The short intracortical inhibition (SICI) and intracortical facilitation (ICF) which represent inhibitory and excitatory activation of interneurons within the motor cortex are thought to probe GABAA-mediated inhibition and glutamatergic facilitation [47].

#### Secondary outcomes: Magnetic resonance imaging (MRI)

MRI scan will use a 3.0-T GE scanner (General Electric, Milwaukee, WI, USA) with an eight-channel phased-array head coil at the Medical Imaging Department of Shanghai Seventh People’s Hospital in China. Two independent MRI scans who are radiologists trained in brain MRI measurements and having ten years of work experience will be performed on each participant at baseline and after the 12-week intervention. Before the MRI evaluation, the scanner will require the participant to rest for 10 min and participants will be asked to stay awake with eyes closed during the entire MRI scan.

Resting-state functional MRI images (Rs-fMRI) will be acquired with the following parameters: TR = 2100 ms, TE = 30 ms, flip angle = 90°, voxel size = 3.125 mm × 3.125 mm × 3.6 mm, 42 axial slices, field of view (FOV) = 200 mm × 200 mm, phases = 230. We also collected high-resolution T1-weighted structural images(T1WI), using a 3D-BRAVO sequence with the following parameters: TR = 8.2 ms, TE = 3.2 ms, flip angle = 12°, FOV = 220 mm 20 mm, Matrix = 256,256, slice thickness = 1 mm [48]. The MRI outcomes include the following: grey matter (GM) density, white matter (WM), subcortical nuclei volumes, cortical thickness; functional connectivity (FC).

The DPARSF (http://rfmri.org/DPARSF) will be used to conduct fMRI data preprocessing [49] and the FSL tools (FMRIB Software Library) will be used to process T1WI structure data analysis [50]. The volumes of neocortical GM, total GM, and WM will be obtained by SIENAX (part of FSL 5.0) [51]. The normalized volumes of subcortical nuclei volumes will be estimated from FMRIB’s integrated registration and segmentation tool (part of FSL 5.0, FMRIB Software Library) [52]. The cortical thickness will be obtained using FreeSurfer.

### Safety and adverse events

Every adverse event during the study will be recorded and reported by the safety officer. All potential risks during the study are listed on the participant’s Informed Consent Form. All of them will complete an adverse effects questionnaire for TMS and MRI after intervention.

### Statistical analysis

Statistical analysis will be performed using IBM SPSS Statistics 25 (http://www.spss.com.hk). Intention-to-treat (ITT) analysis will be used to analyze the result which means the last observation will be used for interpolation when data missing. Continuous variables will be described as mean ± SD for normal distributions, or median and inter-quartile ranges for non-normal distributions, while categorical variables will be described as frequency. For continuous variables that meet the assumptions of a normal distribution and homogeneity of variance, we will use the Two-way of variance with repeated measures; the Wilcoxon test will be used if not. A chi-square test will be used for categorical variables. Pearson correlation coefficient will be performed to detect the correlation between the primary outcomes (VAS, TQPEAK) and secondary outcomes (MEP, FC). When analyzing data obtained by repeated measurements, we will use two-tailed multivariate analysis of variance. The results will be considered statistically significant when the P value is less than 0.05. The post hoc comparisons will be performed by the Bonferroni correction for multiple comparisons if necessary.

### Patient and public involvement

The initial study idea was conceived by the study team. The patients with KOA, physiotherapists, orthopedist and experts from Shanghai Association of Rehabilitation Medicine took part in preparation of the proposal with face-to-face interviews. In addition, the study protocols also will be modified and supplemented utter concerns not addressed in a draft proposed at the time based on their feedback, to ensure the safety and applicability of the intervention. Furthermore, standardized protocol, technical operations, and clinical applications will also be disseminated through Shanghai Association of Rehabilitation Medicine.

## Discussion

KOA is classified as a peripheral joint disease, but changes in sensation-movement and pain are mediated by the central nervous system. Rasch et al.[53]proved that the muscle weakness caused by osteoarthritis is related to decreased joint function and increased pain, and that quadriceps training can increase muscle strength and relieve joint pain. Patients with KOA often exhibit strength deficiencies [1] and altered neuromuscular control [54] compared to those without KOA, which may result in suboptimal load dissipation within the knee. Though the motor output and pain changes resulting from KOA have been well-studied, the central contribution to muscle activity is less well known. In this protocol, we design four groups to prove the therapeutic role of high-frequency rTMS combined with quadriceps strength training compared to quadriceps strengthen training alone in KOA patients with 12-week intervention and explore changes of brain volume and function connection before and after treatment.

There are only two studies to prove the treatment effect when combined noninvasive brain stimulation (tDCS) with exercise has been publicized [55, 56]. However, the design of the trial was inadequate, which used inappropriate randomization procedures had a small sample size. And the evaluation is only limited to the change of pain in KOA. Thus, we designed a stricter study to explore the dual clinical effects of reducing pain and muscle strength when the neuromodulation of the M1 with high-frequency rTMS combined with quadriceps strength training in KOA. In addition, to the best of our knowledge, this will be the first single-blind study on rTMS in KOA. Moreover, we will also analyze the correlation measurements between the primary outcomes (VAS, TQPEAK) and secondary outcomes (MEP, FC). Furthermore, the study’s exclusion criteria are relatively strict which will exclude many patients. In this case, it is possible to ensure that our experimental results have a certain degree of accuracy and validity.

We recognize this study also has limitations. First, the blinding cannot be performed for the participants and the study operator, because of the visibility of the quadriceps strength training intervention, which might increase the risk of detection bias during the study’s implementation. Second, the exercise training in this protocol is only focuses on quadricep strengthening and not strengthening the whole kinetic chain. Although quadriceps weakness is closely related to the occurrence and development of KOA, strengthening the whole kinetic chain may have greater rehabilitation benefits for KOA than alone quadriceps strengthening. Third, account for the absence of direct evidence on rTMS for improve pain of KOA, we incorporated the effect size of rTMS in alleviating pian of the patients with fibromyalgia in sample size calculation [24]. While there are distinctions between the two types of disease, they both involve central sensitization.

In conclusion, the study will provide evidence to show whether there are synergistic effects following High-Frequency rTMS act on M1 combined with quadriceps strength training and clarify the mechanism of action. The study will help provide rehabilitation prescription recommendations for people with KOA.

### Study status

This trial is currently in the preparation phase. Participant recruitment will be started in October. 2023 and is expected to end in December.2023. The version of this protocol is the 1st version and the completion time in Feb. 2023.

## Data Availability

The datasets will be generated and analyzed during the current study are available in the Clinical Trial Management, http://www.medresman.org. We will train all researchers which will record data in case report forms (CRFs) and sign them to ensure data quality. Any changes made on the CRF need to be indicated with a reason and date. Data collection and entry is performed by blinded and independent research assistants. Data storage will be in accordance with the protocols for maintaining security and privacy of data and will be protected by password.

## Abbreviations

KOA: Knee osteoarthritis
rTMS: Repetitive transcranial magnetic stimulation
SPIRT: Standard Protocol Items for Randomized Trials
WOMAC: Western Ontario and McMaster Universities Osteoarthritis Index
Rs-fMRI: Resting state functional MRI images
CRFs: Case report forms
VAS: Visual analogue scale
KOOS: Knee Injury and Osteoarthritis Outcome Score
SF-36: 36-Item Short-Form Health Survey
MRI: Magnetic resonance imaging
ITT: Intention-to-treat
